# Editorial: Paradigm shifts and innovations in cellular neuroscience

**DOI:** 10.3389/fncel.2025.1644329

**Published:** 2025-07-14

**Authors:** Enrico Cherubini, Arianna Maffei, Egidio D'Angelo, Dirk M. Hermann, Marie-Ève Tremblay, Christian Hansel

**Affiliations:** ^1^European Brain Research Institute, Rome, Italy; ^2^Stony Brook University, Stony Brook, NY, United States; ^3^University of Pavia, Pavia, Italy; ^4^Department of Neurology, University Hospital Essen, University of Duisburg-Essen, Essen, Germany; ^5^University of Victoria, Victoria, BC, Canada; ^6^University of Chicago, Chicago, IL, United States

**Keywords:** neurotransmitter release, neuronal ensembles, synaptopodin, primary visual cortex, HiPSCs, focal cortical dyslasia type II, tuberous sclerosis, dendritic integration

In recent years, significant advances in the field of Cellular Neurosciences have contributed to translating basic science into ways to ameliorate diseases affecting the nervous system that carry high economic and social burdens such as neurodevelopmental and neurodegenerative disorders. This has been made possible by emerging technologies such as machine learning and artificial intelligence, humanized mouse and human iPSC models, imaging innovations, brain-computer interfaces, non-invasive brain stimulation, gene editing, identification of biomarkers for drug discovery, etc.

The aim of this Research Topic is to understand the paradigm shifts that have shaped and continue to shape Cellular Neuroscience. This Research Topic includes six Reviews, one Opinion, and four Research Articles.

## Reviews

According to the seminal work at the neuromuscular junction by Del Castillo and Katz (1954), transmitter release occurs in packets of relatively constant size (quanta), which are equal to the content of a single vesicle fused to the presynaptic membrane. Fusion is favored by a complex network of mutually interacting proteins including synaptotagmin. Fesce reports how, with the advent of optogenetics, it has been possible to demonstrate that transmitter release can occur also through the transient opening of a pore between the vesicle and the plasma membrane, without the need for the vesicle to completely fuse with the latter (Harata et al., 2006), as already suggested by Bruno Ceccarelli, who called this event “kiss and run.”

Long lasting, activity-dependent changes in synaptic strength such as those occurring in Long Term Potentation (LTP) are thought to be the cellular correlates of learning and memory (Bliss and Lomo, 1973). Here, Hansel and Yuste suggest that activity-dependent increases in neuronal excitability can recruit neurons into ensembles and maintain them active. They propose *a permissive gate model* by which the enhanced excitability facilitates ensemble integration by converting subthreshold into supra-threshold connections and by promoting the propagation of dendritic potentials toward the soma, thus allowing to enhance the EPSP/spike coupling. This cellular plasticity mechanism can take place in the absence of LTP and thus leaves the synaptic weight distribution unchanged.

Learning and memory processes are associated with morphological modifications of dendritic spines (Bourne and Harris, 2007) which are recognized as the loci of synaptic plasticity expression. Wu et al. highlight recent findings on the functional role of synaptopodin, an actin-associated protein found in a subset of dendritic spines of telencephalic neurons, in various forms of Hebbian synaptic plasticity where it plays a central role in regulating postsynaptic calcium dynamics.

Of particular interest are synaptic plasticity processes occurring in the primary visual cortex (V1), which, as demonstrated by anatomical and molecular studies, develop over multiple time windows, from the first trimester to aging (Siu and Murphy, 2018). Murphy and Monteiro provide an overview of human primary visual cortex development, highlighting the molecular mechanisms regulating the expression of glutamatergic and GABAergic receptors involved in V1 Excitatory/Inhibitory balance and experience-dependent plasticity, including the late shift of GluN2A/GluN2B balance, consequent to the loss of GluN2B subunits in adulthood (Siu et al., 2017).

Emerging technologies such as human induced pluripotent stem cell (hiPSCs) are poised to play a crucial role in identifying the cellular and molecular mechanisms underlying genetic neuropathologies such as neurodegenerative diseases and epilepsy. Toward a precision/personalized medicine, Farahani et al. highlights how hiPSCs derived from somatic cells can produce various neuronal cell types in which non-neuronal immune cell types like microglia can be incorporated to develop new therapeutic tools to prevent and treat these disorders. The use of machine learning/artificial intelligence and quantitative neuroimaging representations would enhance precision by integrating hiPSC-neuronal models with patients' biophysical data (Vo et al., 2024).

Molecular analysis of hiPSC from pediatric patients affected by Focal Cortical Dysplasia Type II and Tuberous Sclerosis has facilitated the identification of several dysregulated genes involved in neuronal migration and differentiation which are responsible for cortical malformations and drug-resistant forms of epilepsy (Afshar Saber and Sahin, 2020; Lu et al., 2024). The review by Zhang et al. focuses on balloon/giant cells (BC/GC), commonly found in these malformations, which are unable to generate action potentials, with special emphasis to their electrophysiological and morphological glial-like properties similar to astrocytes. BC/GC express a range of glial markers, such as GFAP, vimentin, and nestin, indicating a heterogeneous population of cells with mixed neuronal and glial characteristics.

## Opinion

In different brain areas, distinct synaptic input converging onto Pyramidal neurons (Pn) show a macroscale distribution across large dendritic compartments. In this Opinion paper, Cupolillo et al. discuss how spatially distributed excitatory and inhibitory signals converge and integrate onto Pn to shape the neuronal output. The authors provide experimental and computational evidence that these events closely rely on the ability of neurons to generate different forms of local dendritic spikes. The impact of clustering and cooperative plasticity among glutamatergic synapses and the specific spatial organization of GABAergic inputs on dendritic branches are key determinants for shaping dendritic excitability.

## Research articles

Transient elevations of intracellular calcium by voltage-gated calcium channels (VGCCs) activated by back-propagating action potentials play a crucial role in dendritic integration (Stuart and Sakmann, 1994). Using ultrafast membrane potential recordings and calcium imaging techniques, Blömer et al., investigated the kinetics of back-propagating action potentials and associated calcium currents in apical dendrites of layer 5 neocortical pyramidal neurons. In addition, using a realistic NEURON model, they clearly demonstrate that large conductance calcium-dependent potassium channels (BK) are rapidly and selectively activated by N type of VGCCs. Activation of these channels at the dendritic level leads to reduced neuronal excitability.

Early sharp waves (eSPWs) are the earliest network activity observed in the developing rodent hippocampus. In neonates, eSPWs lack high frequency oscillations, called ripples, characteristic of adult SPWs (Leinekugel et al., 2002). Using silicon probes electrodes to record neuronal activity in deep and superficial layers of neonatal medial entorhinal cortex (MEC), Shipkov et al. found that eSPWs are primarily driven by layer 2/3 inputs of the MEC, triggered, *via* sensory feedback, by myoclonic movements. These findings contrast previous results from adult animals, showing that SPWs originating from the hippocampus spread to the entorhinal cortex, thus contributing to memory consolidation.

Recent studies highlighted the physiological role of the ventromedial nucleus of hypothalamus (VMH) in controlling energy and glucose homeostasis (Choi et al., 2013). Using a previously generated BAC transgenic line expressing Cre recombinase under cholecystokinin (CCK) promoter and pharmacological tools, Eftychidis et al. investigated the role of CCK containing neurons, highly expressed in VMH, in regulating food intake, body weight and glucose homeostasis. They found that silencing CCK neurons with DREADDS, or removing them with diphtheria toxin, resulted in increased feeding behavior. Therefore, this approach unveiled new potential targets for obesity treatment.

Finally, Girasole et al. used a nanomotion sensor to monitor, at micro and nanoscale level, the interaction-dependent movements between two clusters of neuroblastoma cells, one of which was growing on a neuro-mechanical oscillator suspended a few hundreds of microns from a Petri dish containing the other. The study reports that cell movements in one compartment are able to influence the other one, located hundreds of microns away. These bidirectional interactions occur *via* acoustic fields produced by vibrations of neuroblastoma cells movements, which, in this way, play a crucial role in cell/cell communication processes.

We thank all those who have contributed to the realization of this Research Topic. We hope that this work will stimulate further studies on new advances in the rapidly growing field of Cellular Neuroscience.
